# Baseline characteristics in female cancer patients with unimproved work status after an outpatient rehabilitation program and health changes during the intervention

**DOI:** 10.1186/s40064-016-2663-x

**Published:** 2016-07-07

**Authors:** Lene Thorsen, Alv A. Dahl, Roy Nystad, Cecilie E. Kiserud, Amy Ø. Geirdal, Sigbjørn Smeland

**Affiliations:** National Advisory Unit on Late Effects After Cancer Treatment, Department of Oncology, Oslo University Hospital, P.O. Box 4953, 0424 Nydalen, Oslo, Norway; University of Oslo, Oslo, Norway; The Outpatient Cancer Rehabilitation Unit, Department of Clinical Service, Oslo University Hospital, Oslo, Norway; Oslo and Akershus University College of Applied Sciences, Oslo, Norway; Division of Cancer Medicine, Oslo University Hospital, Oslo, Norway

**Keywords:** Female cancer patients, Unimproved work status, Health-related quality of life, Outpatient rehabilitation

## Abstract

**Purpose:**

To improve work ability and health-related quality of life (HRQOL) cancer patients were offered a “Rapid-Return to Work” program. However, several patients did not improve their work status after completing the program. The first aim of this study was to identify the proportion of patients with unimproved work status 6 months after the program (follow-up). The second aim was to identify baseline characteristics associated with unimproved work status and the third aim to measure changes in HRQOL from baseline to follow-up in the unimproved compared to the improved group.

**Methods:**

The program consisted of patient education, group discussions and physical activity during a full day weekly for 7 weeks. All patients completed a questionnaire at baseline and follow-up, covering demographic-, cancer-related-, co-morbidity and lifestyle variables, HRQOL (EORTC QLQ-C30) and fatigue (Fatigue Questionnaire).

**Results:**

106 female cancer patients completed the program and responded to the follow-up. Thirty-six percent had unimproved work status. Patients in the unimproved group more frequently were in paired relations and had more fatigue at baseline than the improved group. Whereas patients in the improved group increased in 14 of 19 HRQOL parameters, the unimproved group increased in seven of these parameters. Both groups experienced improvement concerning fatigue.

**Conclusion:**

After the program more than one third of the participants did not improve their work status. Patients in paired relations and with more fatigue at baseline were more likely to have unimproved work status. Those within the unimproved group experienced less improvement in HRQOL parameters during the program than those in the improved group.

## Background

Among Norwegian women diagnosed with cancer in 2014 approximately 2/3 were within working age (18–67 years old) (Cancer Registry of Norway 2015). To be part of the work force is beneficial for health (van der Noordt et al. 2014), and being unable to return to work (RTW) after treatment, frequent and prolonged sick-leaves, or reduced work ability, may therefore have a negative impact on female cancer patients and their families. There are several reports on long-term sick-leave in women diagnosed with breast cancer (Hedayati et al. 2013), while there is less data on gynaecological cancer. Five groups of factors are considered relevant for RTW after cancer (Spelten et al. 2002; Mehnert 2011): national legislation and regulations and economic factors, work-related, demographic, disease related, and personality-related factors.

In order to promote health and work capacity in cancer patients, several rehabilitation programs have been introduced during the last decades with focus on RTW. Based on 18 studies with 1652 cancer patients, a Cochrane review concluded that “moderate quality evidence show that employed patients with cancer experience RTW benefits from multidisciplinary interventions compared to care as usual” (de Boer et al. 2011).

To preclude long-term sick-leave of patients, the Norwegian government recently initiated a “Rapid-Return to Work” (R-RTW) program. At the Department of Oncology, Oslo University Hospital (OUH) an R-RTW program was established in 2009 for outpatients who had finished their cancer treatment but were still on sick-leave or felt unable to return to work. In this study we included 106 female cancer patients who completed the R-RTW program and responded to follow-up assessments 6 months later.

The first aim was to identify the proportion of female patients with unimproved work status 6 months after termination of the program. The second aim was to identify demographic-, disease- and health related characteristics at baseline associated with unimproved work status at follow-up, and the third aim was to measure changes in health-related quality of life (HRQOL), fatigue and physical activity after completing the R-RTW program for patients with unimproved and improved work status.

## Methods

### Patient recruitment

Between 2009 and 2012 female cancer patients treated at the Department of Oncology, OUH, were informed about the R-RTW program by nurses at the outpatient clinics and radiotherapists at the radiotherapy department. When recruiting patients to such studies it is necessary with close cooperation with the medical staff at the hospital. We informed and reminded the staff upfront and during the study period, but not all eligible patients were informed and invited. The staff might have been reminded of the study when patients brought up limitations in work life as a subject for discussion, which in next hand led to information and invitation to participate in the study. Therefore, the study might include a self-selected sample, not necessarily representative for all patients on sick-leave or perceiving themselves in need for sick-leave.

Patients were eligible if they fulfilled five criteria: (1) being within work age (18–67 years); (2) were on sick-leave or had full-time work, but perceive themselves at potential risk for not being able to continue to work full-time and therefore in risk of immediate sick-leave; (3) having recently completed their primary treatment (radiotherapy and/or chemotherapy); (4) being capable to complete all the components of the R-RTW program; (5) being in a curative phase of their malignancies. Data from a sub-sample of 50 eligible women with breast and gynecological cancer have previously been presented in a comparative study of corresponding patients treated in an inpatient R-RTW program (Oldervoll et al. 2014).

### The Rapid-RTW program

The two explicit goals of the program were to improve the work ability and increase HRQOL of the participants. The program was offered outpatient, a full day weekly for 7 weeks. At the start and end of the program, each patient had a consultation with a social worker focusing on individual goals for the program period. Each day the program started with a *patient education* session for 2 h. These sessions covered topics related to cancer treatment, side effects, partnership and sexuality, economy and work situation, nutrition, physical exercise and coping strategies. Relevant health professionals led the sessions. The patient education was followed by 1-h *group discussion* of the topic presented. After lunch the participants performed *physical activity*, such as Nordic walking, water gymnastics, resistance training or yoga/relaxation for 60–120 min, led by a physiotherapist.

### Assessments

The patients completed the same questionnaire at the beginning of the program (baseline) and at 6 month after the R-RTW program (follow-up).

#### Work status

Self-reported work status was assessed with a single question with four response alternatives: full-time work, part-time work, on sick-leave, and work assessment allowance. Change in work status from baseline to follow-up was categorized into (a) improved work status [work status at a higher level at follow-up compared to baseline], or (b) not improved work status [work status at the same level or less at follow-up compared to baseline].

#### Demographic, disease- and health-related variables

*Demographic* variables were self-reported and included age at survey, being in paired relations (yes/no), having children living at home (yes/no) and level of education (≤13/>13 years). *Cancer*-*related* variables were retrieved from the medical records and included months since diagnoses, cancer diagnoses (breast, gynecological, lymphoma and oesophagus) and treatments modalities (surgery, chemotherapy, radiotherapy or combinations). *Co*-*morbidity and lifestyle* variables were self-reported and included obesity (body mass index ≥30), somatic co-morbidity (any diagnosis of heart disease, asthma, diabetes or stroke) and musculoskeletal co-morbidity (osteoporosis, arthrosis, fibromyalgia, or any other chronic disease of the bone, joints, or muscles), daily smoking (yes/no) and level of physical activity. *Physical activity* was measured by three questions concerning frequency, intensity and duration of performed activity last week. The responses are weighted as the Physical activity index with a range from 0 (no activity) to 15 (maximum activity) according to the algorithm developed by Kurtze et al. (2008).

*HRQOL* was assessed by the European Organization for Research and Treatment Core Quality of Life Questionnaire (EORTC QLQ-C30) (Aaronson et al. 1993). The EORTC contains nine multi-item scales including five functional scales [physical (PF), role (RF), emotional (EF), cognitive (CF), and social (SF)], three symptom scales (fatigue, nausea/vomiting and pain) and a global health and quality of life scale (GQL). Six single-item symptoms are also included as well as financial problems. The raw scores were linearly transformed to 0–100 scales. Lower scores represent worse function and lower symptom levels. In this sample the internal consistency of the EORTC subscales showed Cronbach’s coefficient alpha value of PF: 0.65, RF: 0.82, EF 0.77, CF 0.51, SF 0.66, GQL 0.84, fatigue 0.89, pain 0.91, and nausea 0.83 at baseline.

*Fatigue* was assessed by Fatigue Questionnaire (FQ) (Chalder et al. 1993). The FQ includes 11 items covering physical fatigue (P-Fat) and mental fatigue (M-Fat). Total fatigue (T-Fat) is the sum of the P-Fat and the M-Fat subscales score. Each item has four response alternatives scored from 0 to 3, giving a total score of 0–21 for P-Fat, 0–12 for M-Fat and 0–33 for T-Fat. Higher scores imply more fatigue. Patients who report a high level of fatigue for more than 6 months are defined as cases with *chronic fatigue.* In our sample the internal consistency of the P-Fat scale was Cronbach’s alpha of 0.84, for the M-Fat scale 0.80, and for the T-Fat 0.85 at baseline.

### Ethics

The Regional Committee for Medical and Health Research Ethics of South-East Norway approved the study, and all participants have signed an informed consent.

### Statistics

Internal consistencies of scales and subscales in EORTC QLQ-C30 and FQ were given as Cronbach’s coefficient alphas. Differences in baseline characteristics between the unimproved group and improved group on continuous variables were analyzed with independent sample t tests and in case of skewed distribution with Mann Whitney U tests, whereas categorical variables were analyzed with Chi square tests. Changes from baseline to follow-up for HRQOL, fatigue and physical activity within the unimproved group and improved group were analyzed with paired sample t tests.

Adjusting for statistically significant baseline differences between the improved and unimproved groups, multivariable logistic regression analyses were used to explore the associations between demographic and health-related independent variables and unimproved work status versus improved (reference) status as dependent variable. The strength of associations was reported as odds ratios (OR) with 95 % confidence intervals (95 % CI). At baseline the Spearman coefficient r was 0.74 between EORTC Fatigue and P-Fat, so only the former variable was used in the multivariate regression analyses.

Data were analyzed using the PASW for Windows (version, 20) software (SPSS, Inc, Armonk, NY). p values <0.05 were considered significant and all tests were two-tailed.

## Results

Between January 2009 and April 2012, 115 eligible patients completed the program and responded to both the baseline and follow-up questionnaires. Patients working full-time both at baseline and follow-up (nine patients) could not improve and were excluded from the analyses. A final sample of 106 female cancer patients entered our analyses.

### Work status to follow-up

At follow-up 38 patients (36 %, 95 % CI 27–45 %) had unimproved work status and 68 patients (64 %, 95 % CI 55–73 %) had improved. Detailed changes in work status from baseline to follow-up are shown in Table 1.

**Table 1:** Change in work status from baseline to follow-up

Status at baseline	Status at 6 month follow-up				
	Full time	Part time	On sick-leave	Work assessment allowance	Total
Full time	NA	*5*	*5*	*0*	10
Part time	***2***	***4***	*1*	*3*	10
On sick-leave	***36***	***26***	*7*	*12*	81
Work assessment allowance	*0*	*1*	*0*	*4*	5
Total	38	36	13	19	106

Italic: improved patients (n = 38), bold italic: unimproved patients (n = 68), underlined: Non-applicable (NA) since patients working full time both at baseline and at follow-up were excluded from further analyses (n = 9)

### Baseline characteristics

The unimproved group had a higher proportion of patients in paired relations (p = 0.03) and a trend of higher percentage of patients who had children living at home (p = 0.05) compared to the improved group (Table 2). No significant differences were observed for other demographic-, cancer-related-, co-morbidity or lifestyle variables between the groups. The unimproved group showed higher levels of fatigue measured by EORTC and P-Fat measured by FQ compared to the improved group (p = 0.01 and p = 0.04, respectively). A difference in financial problems of borderline significance between the groups was also observed (p = 0.06) (Table 2).

**Table 2:** Baseline characteristics among patients with unimproved versus improved work status

	Improved *(N* = *68)*	Unimproved *(N* = *38)*	p value
*Demographic variables*			
Age at survey, years (mean SD)	50.0 (7.8)	46.7 (9.2)	0.13
In paired relations (n %)	48 (71)	34 (90)	*0.03*
Having children living at home (n %)	26 (38)	22 (58)	0.05
High level of education (>13 years) (n %)	37 (54)	26 (68)	0.16
*Cancer*-*related variables*			
Months since diagnosis (mean SD)	15.5 (5.6)	16.2 (5.4)	0.55
Cancer diagnoses (n %)			
Breast	42 (62)	22 (58)	0.56
Gynaecological	21 (31)	12 (32)	
Lymphoma	4 (6)	3 (8)	
Oesophagus	1 (1)	1 (2)	
Treatment modalities (n %)			
Surgery	56 (82)	30 (79)	0.67
Chemotherapy	50 (74)	29 (76)	0.75
Radiotherapy	50 (74)	29 (76)	0.75
Combinations	28 (41)	17 (45)	0.72
*Co-morbidity and lifestyle variables*			
Being obese (BMI ≥30) (n %)	12 (18)	3 (8)	0.17
Somatic comorbidity (n %)	7 (10)	3 (8)	0.69
Musculoskeletal comorbidity (n %)	13 (34)	19 (28)	0.50
Daily smokers (n %)	4 (6)	5 (14)	0.20
Physical activity index (mean SD)	5.3 (1.8)	5.1 (1.7)	0.54
*EORTC QLQ-C30 functions (mean SD)*			
Physical functioning (PF)	79.7 (14.9)	75.7 (14.5)	0.21
Role functioning (RF)	54.0 (27.0)	46.1 (30.1)	0.08
Emotional functioning (EF)	73.9 (16.7)	68.9 (21.2)	0.31
Cognitive functioning (CF)	75.4 (18.4)	71.9 (19.4)	0.25
Social functioning (SF)	61.7 (21.9)	57.9 (25.3)	0.53
Global health and quality of life (GQL)	60.7 (17.3)	55.3 (16.5)	0.09
*EORTC QLQ-C30 symptoms (mean SD)*			
Fatigue	45.6 (24.0)	58.2 (26.4)	*0.01*
Nausea/vomiting	5.6 (10.6)	8.8 (13.3)	0.11
Pain	30.1 (28.3)	32.5 (30.9)	0.68
Dyspnea	26.4 (26.3)	22.8 (24.6)	0.53
Sleep problems	37.8 (34.8)	34.5 (27.4)	0.50
Appetite loss	11.9 (22.2)	15.8 (26.6)	0.40
Constipation	18.7 (26.9)	18.4 (31.7)	0.92
Diarrhea	17.2 (31.7)	18.4 (26.5)	0.68
Financial problems	7.5 (18.2)	15.8 (29.8)	*0.06*
*Fatigue Questionnaire*			
Physical fatigue (P-Fat) (mean SD)	13.0 (3.4)	14.4 (3.2)	*0.04*
Mental fatigue (M-Fat) (mean SD)	6.1 (1.8)	6.0 (1.9)	0.93
Total fatigue (T-Fat) (mean SD)	19.0 (4.6)	20.5 (4.2)	0.13
Chronic fatigue (n %)	19 (29)	13 (35)	0.73

Italic: p values <0.05 were considered significant

*SD* standard deviation, *n* number, *BMI *body mass index, *EORTC QLQ-C30* European Organization for Research and Treatment Core Quality of Life Questionnaire-C30

After adjusting for significant baseline differences between groups, being in paired relationship and fatigue measured by EORTC QLQ-C30 remained significantly associated with unimproved work status in the multivariable analysis (Table 3).

**Table 3:** Baseline variables associated with unimproved work status (improved as reference) in multivariate analyses

	Multivariate analyses		
	OR	95 % CI	p value
Paired relationship	0.23	0.07–0.80	*0.020*
EORTC Fatigue^a^	1.02	1.01–1.04	*0.012*

Italic: p values <0.05 were considered significant

*EORTC Fatigue* Fatigue symptom scale in European Organization for Research and Treatment Core Quality of Life Questionnaire-C30

^a^Correlation between EORTC fatigue and FQ Physical fatigue is r = 0.75, and only the first variable was used in multivariate analysis

### Changes from baseline to follow-up

Whereas all EORTC mean function scores increased significantly from baseline to follow-up in the improved group, EF, CF and SF did not increase significantly in the unimproved group (Table 4). The EORTC mean fatigue scale scores decreased significantly in both groups, but the mean EORTC nausea, pain, dyspnea, sleep problems and appetite loss scores only decreased significantly in the improved group. In contrast, the levels of financial problems and physical activity index improved significantly in the unimproved group, while these variables hardly changed in the improved group. Both mean P-Fat and T-Fat scores were significantly reduced in both groups.

**Table 4:** Estimated mean change in HRQOL, fatigue and physical activity from baseline to follow-up among patients with unimproved versus improved work status

	Changes within the improved group *(N* = *68)*		Changes within the unimproved group *(N* = *38)*	
	Mean change	p value	Mean change	p value
*EORTC QLQ-C30 functions (mean SD)*				
Physical functioning (PF)	8.5	*<0.001*	6.1	*0.022*
Role functioning (RF)	25.8	*<0.001*	17.6	*<0.001*
Emotional functioning (EF)	9.0	*<0.001*	5.4	0.06
Cognitive functioning (CF)	5.4	*0.017*	3.9	0.36
Social functioning (SF)	18.4	*<0.001*	8.8	0.09
Global health and quality of life (GQL)	14.1	*<0.001*	7.2	*0.035*
*EORTC QLQ-C30 symptoms (mean SD)*				
Fatigue	−17.9	*<0.001*	−18.3	*0.001*
Nausea/vomiting	−2.7	*0.040*	−2.3	0.34
Pain	−9.7	*0.003*	1.3	0.79
Dyspnea	−14.9	*<0.001*	−7.2	0.15
Sleep problems	−11.6	*0.002*	− 0.9	0.87
Appetite loss	−9.0	*0.002*	−5.4	0.25
Constipation	−5.6	0.15	1.9	0.69
Diarrhea	−4.0	0.15	−1.9	0.76
Financial problems	1.5	0.58	10.8	*0.012*
*Fatigue Questionnaire (mean SD)*				
Physical fatigue (P-Fat)	−3.1	*<0.001*	−3.3	*<0.001*
Mental fatigue (M-Fat)	−0.4	0.08	−0.2	0.48
Total fatigue (T-Fat)	−3.5	*<0.001*	−3.5	*<0.001*
Physical activity index	0.2	0.33	0.7	*0.022*

Italic: p values <0.05 were considered significant

*EORTC QLQ-C30* European Organization for Research and Treatment Core Quality of Life Questionnaire-C30, *SD* standard deviation

## Discussion

In our sample, 36 % of female cancer patients going through an outpatient R-RTW program did not improve their work status. In multivariate analyses, being in paired relationship was the only demographic characteristic associated with unimproved work status, but univariate analyses also showed a trend toward significance that having children living at home also was associated with unimproved work status. More fatigue at baseline was also significantly associated with unimproved work status. In general, those with unimproved work status experienced less improvement in HRQOL measures during and after the R-RTW program than those with improved work status.

In a systematic review of four intervention studies in breast cancer patients, Hoving et al. (2009) concluded that 75–85 % returned to work after a rehabilitation program. However three of the studies did not include a comparison group so it is unclear whether these proportions would have been lower without the interventions. After a Norwegian inpatients rehabilitation program designed for women with breast cancer, 78 % (36 out of 46) of the participants returned to work (Fismen et al. 2000). The proportions of improvement observed in this study did not differ significantly to the proportion of patients with improved work status reported in this study (p = 0.09).

In a Cochrane review three controlled trials examining the effect of multi-dimensional rehabilitation interventions on RTW were identified (de Boer et al. 2011). The authors concluded that moderate evidence supported that these interventions led to higher RTW rates compared to standard care, but none of these studies examined variables associated with non-RTW. Observational studies, however, showed that non-RTW cancer survivors are characterized by older age, lower education, female gender, economic problems, more fatigue, and higher stage disease (Mehnert 2011). Fatigue and economic problems were the only factors confirmed in our study.

We found that the proportion of women in paired relationships was significantly higher in the unimproved group. A near to hand explanation is that women without a partner are forced by their poorer economy to improve their work status, while the others are supported by the income of their partners. In a meta-study of ten qualitative studies of breast cancer survivors economic pressure was reported as an important factor for RTW in such women (Banning 2011). Interestingly in our improved group, the mean score of economic problems hardly changed from baseline to follow-up, while a significant increase of the mean score was observed among the unimproved women. These results might be related to care for children living at home, that was higher among the unimproved group. This finding showed a trend toward significance that probably would have reached significance if our study had more statistical power.

In contrast to other studies of women with breast cancer (Johnsson et al. 2007), we observed no effect of treatment modalities on work improvement, a result that was supported by the Danish national study (Carlsen et al. 2013). Low level of education in breast cancer survivors has also been associated with lack of RTW (Carlsen et al. 2013), but that result was not supported in our sample.

Since the groups did not differ much at baseline, we assume that improvements in more EORTC functions and symptoms at follow-up in the improved group might be due to better use of the rehabilitation program. We therefore consider that other factors related to the unimproved group might be reasons why they had less use of the program. We suggest that the differences in usefulness and ability to learn from the program because a lower level of motivation among women in paired relationship due to less economical consequences, and more fatigue might have reduce the ability to participate actively in the program. However, we can not ignore that lack of improvements in the medical conditions in the unimproved group might be the reason for inability to return to work.

Interestingly, only the unimproved group had significant improvement of physical activity. An explanation might be that their baseline levels were lower. As many rehabilitation programs demand inpatient status, our R-RTW program is less expensive and demand of health care personnel only involved in the program.

In Norway sick-leave with 100 % of income are granted for up to 1 year, the unemployment rate is three percent for women, and 38 % of women work part-time (5. Arbeidskraftundersøkelsen www.ssb.no/aku/ 14.04.15). Health care such as cancer diagnostics and treatment are close to free of charge. Hence, our findings must be related to the specific Norwegian labor market, social benefits, and health care system and the generalizability are limited to countries with other factors relevant for RTW.

### Strengths and limitations

A strength of our study was a well-defined and broad daily program of intervention covering both information, group sessions and physical activity as well as individual sessions at the beginning and end of the study. Another strength was the use of well-established self-rating instruments with good psychometric properties. Weaknesses of this study were lack of a relevant comparison group and the small sample in the unimproved group, which might implies lack of statistical power, and an obvious risk of type II statistical errors.

## Conclusion

In this outpatient R-RTW program including a full day weekly activity for 7 weeks, more than one third of female cancer survivors did not improve their work status. Patients in paired relations and with fatigue before starting the program were more likely to have unimproved work status. Both groups showed progress within fatigue, physical- and role functioning and GQL, but the unimproved group experienced no improvement in emotional-, cognitive- and social functioning, as well as more financial problems during the R-RTW program. Our intervention is relatively cheap in resources and manpower and the effect of such rehabilitation intervention should be confirmed in a randomized controlled trial.
